# QUANTIFYING ASSOCIATIVE AND ATTENTIONAL INFLUENCES ON MEMORY FOR EVENTS

**DOI:** 10.1093/geroni/igae098.2657

**Published:** 2024-12-31

**Authors:** Alan Kersten, Kevin Darby, Megan Smithwick, Julie Earles

**Affiliations:** Florida Atlantic University, Boca Raton, Florida, United States; Florida Atlantic University, Boca Raton, Florida, United States; Florida Atlantic University, Boca Raton, Florida, United States; Florida Atlantic University, Boca Raton, Florida, United States

## Abstract

Aging is associated with declines in multiple aspects of the human memory system, including hippocampally-mediated associative abilities and frontally-mediated executive abilities. It is important to distinguish these different impacts of aging in order to identify possible causes of memory decline (e.g., Alzheimer’s disease vs. vascular dementia). In the present research, we used computational modeling to quantify the contributions of different cognitive processes to performance on a test of event memory, administered to 195 young adults and 195 healthy older adults. Participants viewed videos of actors performing simple actions and were later tested on memory for who did what. Participants in different conditions saw varying numbers of actors and experienced different levels of distraction, providing differential opportunities for executive involvement. A multinomial processing tree analysis was used to quantify different aspects of memory performance, including action memory, actor memory, and binding of actors with actions. Distraction impacted both actor memory and action memory, suggesting that distraction interfered with the deployment of attention to these event features. The largest impact of aging was on binding of actors with actions, especially when many different actors performed the actions. Distraction also impacted binding when only two actors performed all of the actions, suggesting that distraction interfered with strategic encoding of actor/action pairings in this condition. The large age differences and absence of an effect of distraction in the many actor condition suggest that this condition may be uniquely sensitive to hippocampally-mediated associative abilities, thus offering diagnostic potential for pathological conditions such as Alzheimer’s disease.

